# Hospital and Institutional News

**Published:** 1914-05-23

**Authors:** 


### HOSPITAL AND INSTITUTIONAL NEWS.

### OUR PRIZE SCHEME FOR EQUIPMENT "TIPS."

The second competition of this series, which we announce on the third page of the cover of this issue, is on bedstead-lifters. There are various types of these on the market, such as the wheeling castor, mounted on a column in the centre of the bottom end of the bedstead, and the detachable lifter for use with the "Lawson Tait" bedsteads. These are described and illustrated on page 219 of the present number. Doubtless, however, many hospitals have their own pattern of bed-lifter, which may be good or bad, whilst many hospital workers may have in their mind an ideal bed-lifter incorporating improvements on existing patterns. All institutional workers are, therefore, asked to co-operate by sending to us a description and photograph either of a bed-lifter in use which their practical experience leads them to think is the best, or a description and sketch of a suggested improvement. The six best designs will in due course be published, and our readers will then be asked to give their opinion as to the order of merit of these six. The conditions of the competition, together with particulars of the prizes which will be awarded, will be found on the third page of the cover. The "bed-lifter" competition closes on June 27.

### DIRECTOR OF RESEARCH UNDER THE INSURANCE ACT.

At the end of the year, it is announced, Dr. Walter Morley Fletcher will leave Cambridge, where he occupies the post of tutor at Trinity College, to take up that of Director of Research under the Insurance Act. Graduating B.A. at Cambridge in 1894, M.B. in 1900, and M.D. in 1908, his work on the functions of muscular tissue has gained him a reputation which also extends to that of organising ability. Mr. E. Harrison will succeed him at Cambridge. It will be recalled that the funds available for research under the Insurance Act have been placed in the hands of a committee, known as the Medical Research Committee, to administer them, of which Lord Moulton is chairman, and on which are Dr. Christopher Addison, M.P., Sir Clifford Allbutt, Mr. C. J. Bond, Mr. Waldorf Astor, Dr. William Bulloch, Dr. Matthew Hay, Dr. F. G. Hopkins, and Colonel Sir W. B. Leishman. The Research Committee is to draft schemes which may include inquiries into matters deemed of special importance, the collection and arrangement of statistics, and so forth, which are to be submitted for approval to the Chairman of the National Health Insurance Joint Committee. The Mount Vernon Hospital was secured for the work of the Research Committee, this giving a clue to the primary idea of the better prevention of tuberculosis, but inevitably growing into the beginnings of a scheme for extending research into other diseases when they become similarly ripe for it—if not in our ignorance, yet in the public admission of it.

### A POOR-LAW MEDICAL OFFICER AND THE USE OF BRANDY.

Dr. W. T. G. Robinson, the medical officer of the Poole Workhouse, has made a spirited protest against the attempts made by some of the members of the local Board of Guardians to interfere with him in ordering stimulants to his patients. In a report to the Board he says: "I am well aware that the question of ordering alcohol is one where conscientious work on my part may land me in difficulties with certain members, whilst trying my best in the interests of the very poor placed in the infirmary. It is astonishing to notice the good a tablespoonful of brandy does them. I have tried some of the substitutes for it; but the patient either refuses to swallow the drug or spits it out, or it acts as an irritant to the enfeebled stomach and is rejected. What may seem economy to some members of the Board, to me as a strictly temperate medical man means an earlier and more painful death to my poor helpless patients, to refuse to let them have a medicine they can retain and one that can be helpful to them." The report met with the approval of a majority of the Board, and a resolution, calling for a return showing the consumption of brandy in the Poole Workhouse now as compared with six years ago, was defeated.

### THE ISOLATION HOSPITAL "GARDENER."

The scope of the work of the hospital "handy-man" was amusingly shown at a recent meeting of the Trowbridge and District Joint Isolation Hospital Committee when the "gardener" applied for an increase of wages. During the discussion that ensued, it was mentioned that the applicant was not a gardener in the full sense of the word. He had to go in and out of the wards of the hospital, he had to go and fetch bedding in connection with tuberculosis cases, as well as fetch and take home again all the patients, apart from doing many things in the hospital which ordinarily would require the services of tradespeople. The application was granted and the wages fixed at 25s. per week. Porters in large institutions will be interested to compare these multifarious duties with their own.

### MALE NURSES IN POOR-LAW INFIRMARIES.

The Wandsworth Board of Guardians has recently decided to engage male nurses for one of their ward blocks instead of women. This decision is one of very great interest. The presence of at least one male nurse on the staff of a Poor-Law infirmary is almost essential. To mention only one instance, the proper nursing of male venereal cases without such an officer is extremely difficult.

In most infirmaries this difficulty is met by making the male receiving wardsman act as nurse for such patients. But, in addition to these special cases, there are many male patients—especially those suffering from diseases of the bladder, old age, and paralysis—who would be much happier and more comfortable if they could be looked after, at any rate partly, by male nurses. In every infirmary there are also male patients, quite apart from those suffering from venereal disease, who are unsuited to be nursed by women. Provision for such cases has been made to a small extent of recent years in the Metropolitan area, and it is now possible for London Unions to send them to the Bow institution of the City of London, where they are attended by men.

### THE SEX QUESTION IN INFIRMARY ADMINISTRATION.

There are three difficulties in the way of the more general employment of male nurses. One is the expense; another, the absence of any system of efficient training; and the third, the administrative difficulties of providing for a staff of combined male and female nurses. As regards the first, although the cost of male nursing is greater, the difference is not so large as to be a very serious matter, and the third, although a real difficulty, is not insuperable. It is the second difficulty—the scarcity of well-trained male nurses—which has influenced the general adoption of female nursing in institutions. Now that the number of women desirous of being trained as nurses is diminishing considerably, the improvement of the training of male nurses, so as to provide an alternative supply, is becoming a matter of importance. At present practically the only training schools for male nurses are the Army and Navy. A few hospitals—notably St. Bartholomew's, the Middlesex, and the National Hospital for the Paralysed and Epileptic —also train male nurses, but the number so trained is very small. Judging by the number of inefficient male nurses one meets with, the training given at all these schools is incomplete, and sadly needs revision and standardisation.

### A NURSING TRAINING SCHOOL FOR MEN.

The establishment of a Poor-Law training school for male nurses is a matter well worthy of consideration. The Bow institution, referred to above, admits male patients only. It receives chronic medical cases, such as bronchitis, heart disease, rheumatism, and paralysis; chronic surgical cases, such as varicose ulcers, and inoperable malignant disease, old people suffering from minor ailments, venereal cases, and men who are deemed for any reason unsuitable for the care of female nurses. A clinic of this description affords just the material for the proper training of a male nurse. The essential qualifications required of him are that he should be able to carry out the treatment ordered in venereal cases, that he should be thoroughly grounded in the theory and practice of aseptic surgery so far as regards the preparation of a patient for an operation and the after treatment of the wound, understand the nursing of a bedridden case so as to prevent bedsores, and know how to pass a catheter with aseptic precautions and wash out a bladder. The patients of the Bow institution would afford the male probationer nurse ample opportunity for obtaining practical knowledge in all these subjects, as well as in the general details of nursing.

### IS THE FUTURE TO NURSING BY MEN?

The formation of a really efficient class of male nurse might lead to some unexpected results. Custom and tradition have led to most men tacitly accepting the services of a woman to nurse them when ill, but there are many men whose instincts revolt against the system and who would much prefer to be nursed when seriously ill by a man who had been properly trained for the work. In the case of Poor-Law infirmaries one may mention as a special instance that it is quite common to find that old men suffering from prostatic enlargements are much happier and more contented with male nurses than with female nurses.

### A PROVINCIAL HOSPITAL GARDEN PARTY.

The garden of the Gravesend Hospital presented a pretty picture on Thursday last week when the members of the Ladies' Association held a small sale of work in order to help provide linen and other necessaries for the children's ward, which, since its institution in 1884, has always been their protégé. The sale was really an echo of the "Olde Village Fayre," which, as The Hospital noted at the time, was successfully organised last year under the auspices of the Association, for it was the means of selling off things left over from that function, the proceeds of which were, of course, benefited considerably. The committee seized the occasion for throwing open the hospital for public inspection, of which privilege many people availed themselves. Taking tea on the lawn to the band of the Dublin Fusiliers, who kindly gave their services, was very popular; indeed, the whole affair was a very happy and enjoyable gathering, and was the means of raising over £60 for the worthy object.

### A MEDICAL MAYOR ON MUSIC.

At the closing concert held at the Roman Promenade, Bath, the medical Mayor, Dr. Preston King, said that in the surroundings necessary for the healing of the sick, and, after all, a great proportion of the visitors to Bath were invalids, music played an important part, and he did not think the medical profession paid enough attention to music. A little music was better than a great deal of medicine. The same sentiments are beginning to permeate the educationalists, and in another thousand years or so we shall perhaps have progressed sufficiently far to put Plato's well-known point of view into practice.

### THE RIVER AMBULANCE SERVICE.

The Metropolitan Asylums Board has decided to adopt the title of "River Hospitals and Ambulance Service" for the Long Reach, Orchard, and Joyce

Green Hospitals and the River Ambulance Service. Now that approval has been given to the regular use of Joyce Green Hospital for fever purposes and of the River Service for the conveyance of fever patients, and, in case it may be necessary in the coming autumn to use the Orchard Hospital for fever purposes, it was deemed advisable to discontinue the present title of "Smallpox Hospitals." Long Reach Hospital and the adjacent pier buildings are now the main accommodation reserved for smallpox cases, and to use the title of "Smallpox Hospitals" to the whole group of hospitals was felt not only to be a misnomer, but was likely to create prejudice against the use of Joyce Green Hospital for fever purposes. Hence the reason for the change in title, which denotes the position of the hospitals and at the same time avoids that reference to smallpox which was felt to be open to misunderstanding.

### THE CONTINUOUS-IMMERSION BATH.

One of the April questions in *The Institutional Worker* section of The Hospital invited particulars as to the best and least expensive type of bath for the continuous immersion of a patient suffering from severe burns. It was certainly a very valuable question, because in this country, at any rate, in voluntary hospitals and Poor-Law infirmaries alike, the problem seems to have met with no easy solution. The question, therefore, was whether the readers of *The Institutional Worker* would be able to supply a solution as important as is the question which occasioned it. This week we are able to supply the answer in *The Institutional Worker*, but on this page of the paper another point must be made. A question of this difficult kind has tested the value of *The Institutional Worker* pretty severely, and the answer now provided proves two very useful things. The first is that through the pages of this section expert advice and experience can be exchanged between institutional workers of every type, one supplementing the deficiencies of knowledge in another; and the second that where, as in the present case, answers and experience are hard to come by, *The Institutional Worker*, from the resources at its disposal, can provide information which is unobtainable in any other way. These facts, once understood, will allow the answer to be studied in its proper light and perspective; the details are given on page 1 of the Special Supplement.

### THE MARRIAGE OF FAITH AND SCIENCE.

The interesting letter on this subject which we published last week from the Chaplain of the Guild of Health, of which, by the way, that energetic gentleman, the Rev. Percy Dearmer, is a member, shows how much genuine study is being given to mental and spiritual healing at the present time. It is remarkable that Mme. Lipinska, the woman doctor, who has since lectured on Suggestion, said, like our correspondent, Mr. Charles Gardner, last week, we sometimes thought of mind and body as two things entirely distinct from each other; but this traditional dualism did not exist. The mind acted directly on the body. Thought was not an occurrence which takes place in an ethereal and intangible world. An idea introduced into the mind followed its own course unknown to us, with the regularity and certainty of an automaton, and finally reappears in consciousness, when our thoughts are far from it. The medical scientist and the modern clergyman, in this case we note, are coming to be at one on this point.

### MENTAL HOSPITALS AND SUGGESTION.

A contribution to the subject, from the point of view of the mental hospitals, was given last week by Dr. Theo. Hyslop, formerly medical superintendent of Bethlem Hospital, at the discussion at the Lyceum Club. The subject being Auto-suggestion, Dr. Hyslop gave a summary of his twenty odd years of experience in mental hospitals. He began by remarking that these institutions necessarily contained a very large, if not actually exclusive, number who had quite passed the borderland of sanity, and added that, therefore, suggestion was of no therapeutic value for them. Only one person, he proceeded, as far as his personal knowledge went, had ever benefited from this type of treatment—namely, a would-be hypnotiser, who was chivied out of the room by his patient, and gained thereby a lot of valuable exercise from the need for escape, which was suggested to him by his patient's behaviour. As to suggestion during sleep, Dr. Hyslop remarked that in normal cases when a person fell asleep the senses gradually sank during the first two hours. Then there was a return towards consciousness, when, if one had had an indigestible meal, one was apt to wake up. Then, again, one sank into deeper sleep and then gradually came closer and closer to consciousness. That was the time when the subconscious activities became most pronounced, and therefore the time *par excellence* to make a suggestion to anyone if you wanted to get anything out of them. What problems for the appeal-organiser are not revealed here!

### THE MILK PROBLEM IN MILITARY HOSPITALS.

It is not often that the Service hospitals give occasion to public reports in the newspapers, but a curious situation arose lately at Haslar. It seems that the General Purposes Committee of the Hampshire County Council received information that separated milk was being delivered in large quantities as "new milk" to Haslar Hospital, with the result that an inspector made a visit to the institution. The samples that he took in the course of delivery were examined by him, and their nature led to the fining of the salesman to the extent of £18 odd apart from costs. But this result apparently did not fill the Admiralty with unmixed gratitude, for they intimated to the County Council that its milk inspector and his activities (whether beneficent or not) were hardly welcome, because they might result in a certain amount of "undesirable and unnecessary overlapping." Overlapping is certainly undesirable, but only one who preferred the letter to the spirit, in short, an official in red-tape (or blue uniform) would consider it "unnecessary" where one of the two authorities responsible has failed apparently to find a defect, later revealed by the other. It seems, in fact, a case rather of "oversleeping" than of "overlapping," and as those who have overslept are not generally in the best of tempers when aroused by a hand however friendly, we may take it that the above is only the Admiralty's human way of stating that they are going to keep wide awake in future.

### SUDDEN ILLNESS OF A LONDON HOSPITAL CHAIRMAN.

Great regret was felt last week at the absence of Mr. Douglas Owen from the annual meeting of the Paddington Green Children's Hospital, of which he is chairman. Sympathy was intensified by the fact that the illness was sudden. Mr. Nigel Hanbury, the treasurer, who took his place, again verified the attitude taken up by the Children's Hospital authorities when the Insurance Act was introduced—namely, that their work would certainly not diminish and almost certainly increase, not in one but in both departments. Both in-patients and out-patients have shown an increase. The latest available reports of Mr. Owen's health state that he is making satisfactory progress.

### MR. A. J. BALFOUR AND ALTON HOSPITAL.

The latest of those many speeches on behalf of the funds and the activities of our voluntary hospitals, which Mr. Balfour has constantly delivered, was one made last week at the first festival dinner on behalf of the Cripples' Hospital and College at Alton. The cure of patients, the education of the practitioner, and research were described by him as the threefold work of the hospitals, and now that the claims of research, in process of time and by persistent work on the part of those interested in it, have become fashionable in the public mind, he took advantage of this awakening to enforce the claims especially of this department. He found a ready support in his audience for this part of his speech, and easily carried them with him in a delineation of the chief aims of the Alton institution, which he reminded those present was to arrest tubercular disease in the bones and joints of children whose whole future would be overshadowed were it not dealt with in time. He endeavoured to point the moral of this by adding that a special institution was required for this work, and described the peculiar subdivision which was deemed essential, adding that the Alton Hospital was a combination, a congeries, of institutions, comprising a hospital, a sanatorium, a convalescent home, with the medical and surgical treatment that no ordinary, meaning general, hospital could supply. We may gratefully take it that the audience felt that they had received as much as they had given; the total amount of money raised by the dinner amounted to £6,775. It is always interesting to hear the claims of those special hospitals which really are doing special work better than it can be done elsewhere—a state of things, as we all know, not wholly represented by all the special hospitals that have sprung into existence, though more or less true of those institutions and types which have survived the vogue out of which they sprang. For this reason double interest attached to this particular dinner and Mr. Balfour's speech.

### A PRACTICAL TRIBUTE TO A HOSPITAL STAFF.

The directors of the County and City of Perth Royal Infirmary have received from Mr. Summers Hunter, of Tynemouth—himself a director of the Newcastle Infirmary—a letter expressing his sincere thanks for the kind and skilful attention given by the surgeons and nurses to his son, who, as the result of a serious motor-cycle accident at Perth, was removed to, and received lengthy treatment at, the institution. With his letter Mr. Hunter forwarded, as an expression of his deep appreciation, a cheque for £50, accompanying the gift with the wish that the amount will be expended for some special purpose at the discretion and to the benefit of the surgeons and the nursing staff in their respective departments.

### TYPHOID AND THE MEXICAN CAMPAIGN.

A report just published of the result of anti-typhoid vaccination in the United States Army during 1913 shows that during that period in the entire Army of over ninety thousand men only three cases of typhoid fever occurred, with no fatalities. The disappearance of the disease is held to be due to the inoculation of all recruits as they enter the Service, and what this result means will be appreciated when the varied conditions under which these men live are realised. About ninety thousand soldiers are scattered over the Philippine Islands, Oahu in the Hawaiian group, Northern China, Panama, Alaska, and the United States proper. The official organ of the American Medical Association recalls the appalling experience of the Spanish-American War, with its record of twenty thousand cases of typhoid in the Army in six months, and remarks that the next campaign in which the Army will participate will be a practical test of typhoid prophylaxis on a large scale. It is confidently expected to prove the value of inoculation and to relieve warfare of one of its most horrible accompaniments.

### WHAT THE SOUTH LONDON HOSPITAL OFFERS TO WOMEN DOCTORS.

Dr. Maud Chadburn, who presided at the second annual meeting of the South London Hospital for Women recently, stated that temporary premises had been secured giving accommodation for eleven or twelve in-patients. The reason of this appears to be that, though Princess Louise laid the foundation-stone of the new hospital on July 1 last, owing largely to the builders' strike the process of building has been delayed. On the other hand, the financial position seems to be satisfactory in the sense that of the £55,000, which was the estimated cost of erecting the new institution, £43,000, or practically four-fifths, has been raised. When the new hospital is completed it will have seventy beds, which will be distributed between four general wards and several private wards. As to the out-patient department, which was opened in April last year at 88 and 90 Newington Causeway, 1,619 patients were treated. Lady Tullibardine's remark at the meeting that she hoped that the training given in the South London Hospital for Women would encourage volunteers for the work of women doctors in India points the way to those sides of medical practice for which women doctors are not merely specially fitted, but also urgently required.

### WORKHOUSE WAGES FOR THE PHYSICALLY UNFIT.

A proposal, made by the Stepney Board of Guardians, to improve the condition of some of those unable to earn their living by the labour of which they are capable, now awaits the sanction of the Local Government Board. The Guardians suggest that they should be empowered to grant a small weekly wage, and special clothing, diet, and other privileges, to girls and young women, chargeable in the workhouse and unfitted for domestic service owing to physical defect, in return for such service as they are capable of rendering. The suggestion is, of course, directly contrary to the old Consolidated Orders and the present Institutions Order. Both Orders direct that the inmates of the workhouse are to be kept employed according to their capacity and ability, and are not to receive any allowance or other compensation in respect of their labour. The only exception made is in the case of those employed upon work of an exceptional or specially disagreeable character, who may be given an allowance of tobacco or snuff. The Stepney Guardians' proposal to vary the strictness of these rules in favour of the small class they define is an admirable one, and will meet with general approval. The class includes the blind, the paralysed, and the crippled, who are not completely incapacitated from work. No class deserves greater sympathy. Many of them are capable of useful work, but are unable to compete with the able-bodied in the labour market. A small wage earned by their work will mean all the difference to them between the feeling of hopeless dependence and the self-respect of those whose labour is worthy of hire.

### DEATH OF AN EX-HOSPITAL ALMONER.

Many sides of institutional life were within the experience of Mr. Robert Gray, who died last week. At the time of his death connected with the Foundling Hospital, of which he had been treasurer since 1892, and an energetic and enthusiastic governor for thirty-eight years, Mr. Gray was as much respected in the City as in the hospital world among the men of his generation. As a Freemason, enthusiastic in this as in all directions, and a Past Master of the Watermen and Lightermen's Company, he was a keen institutional worker in all the various organisations of the craft. Hospital men, however, will turn with more general interest to his work for St. Bartholomew's Hospital, where he held the post of almoner for four years. As governor of Charing Cross Hospital, as well as of the Foundling Charity, hospital work meant for him a life, a mode of living, in which all the best of his character and interests found their highest expression. Such men, it is always important to remind ourselves, are the peculiar glory of the voluntary hospital system, for though all super-selfish work is the product of fine men, whether it happens to be in addition a man's bread and butter or not, yet a clear thinker of modern times was correct in stating that it is only in voluntary associations that man is fine. The paid officer, notwithstanding all the essential business side of his work, is working not for commercial profit but for sick people, and the few black sheep who are incapable of this disinterestedness are never the men who win, or are capable of holding, the prize posts of hospital administration.

### RUBBER FLOORING FOR GUY'S HOSPITAL.

A rubber floor, the gift of the Rubber Growers' Association, which has been accepted by the governors of Guy's Hospital, will be laid in one of the wards of that institution by the date of the Rubber Exhibition, which is to be opened in September. It will be remembered that a similar gift to the Edinburgh Royal Infirmary was announced in these columns on March 7 last.

### PERSONAL SERVICE IN BRISTOL.

The Bristol General Hospital's committee has elected as a vice-president of the institution Mr. Albert M. Fry, nephew of its late president, Mr. Joseph Storrs Fry. The former was closely associated with his uncle in business for a term bordering upon forty years, for Mr. Albert M. Fry quitted Clifton College, where he had received his education under Dr. Percival, the present Bishop of Hereford, in 1874, and straightway began his business career in the firm of Messrs. J. S. Fry and Sons, where he took a position in the management of the factory department, for which a vigorous constitution, fostered by football playing (he was in the Gloucester County Football Fifteen), boating (he was for many years an active member of the Clifton Rowing Club), riding, and golfing, stood him in good stead. For some little time he has retired from active management. Bristol may be proud of the fact that representatives of her largest industries are ready to give their brains, as well as their money, in personal service for her hospitals and institutions.

### PANEL CHEMISTS AND THE DRUG FUNDS.

The returns for the first quarter of the new Insurance year indicate that in a number of districts there has been a considerable increase in the quantity of drugs prescribed, and suggest the probability that in some areas in which the drug funds were sufficient to pay the bills in full last year there will be a deficit at the end of the present year. In view of the situation the Local Associations

Executive Committee of the Council of the Pharmaceutical Society has resolved to take immediate steps to secure a complete report of the facts in all the deficiency areas as a preliminary to initiating effective national action. Among pharmacists the view appears to be held that their bills will eventually be paid in full. It is interesting to note that the pharmacists have set up a small committee to meet a committee appointed by the British Medical Association in connection with the revision of the drug tariff and collateral matters.

### THE GROWTH OF THE PHARMACEUTICAL SOCIETY.

The report of the Council of the Pharmaceutical Society, which was presented at the seventy-third annual meeting held on Wednesday, May 20, shows that the Society is much stronger numerically than it has ever been, the number of members being over 8,000. In every department of the Society's work the influence of the Insurance Act has been felt, and numerous changes have had to be made in the organisation of the Society, both at headquarters and in the provinces to cope with the extra amount of work entailed. About sixty new associations were formed during the year under review, and every part of England and Wales is now covered by a local association. Referring to the dropping of Latin from the compulsory part of the Preliminary Examination syllabus, the report stated that the effect of this decision of the Council was almost immediately a large increase in the number of persons applying for registration as apprentices or students. A knowledge of Latin will still be required in the Minor Examination so far as it relates to the translation and interpretation of prescriptions. During last year 822 candidates presented themselves for the Qualifying Examination in England and Wales. This is the first time for many years that it has been possible to report an increase in the number of entries, and, as the report stated, " is an additional sign that the turning point has been reached, and the commencement of a steady increase in the number of persons entering the calling of pharmacy." A further improvement in the knowledge and training of the candidates entering for this examination is reflected in the increase in the number of those successful. On the whole, the report reflected a more prosperous condition of pharmacy, and, provided the difficulty which is being experienced by panel chemists in some areas of obtaining full payment of their accounts for Insurance dispensing can be overcome, the outlook seems very hopeful.

### LATEST ENLARGEMENT OF POOR-LAW AREAS.

We are gratified to see that the policy of enlarged Poor-Law areas, which we have consistently advocated in these columns, is being slowly adopted by the Local Government Board. The latest instance is that of Manchester, where the Board has just decided to amalgamate the three Poor-Law Unions of the city—the Manchester Township, South Manchester, and Prestwich. These three Unions each possess an infirmary, two of which have not yet been separated administratively from the workhouse. These non-separated infirmaries are of immense size. Manchester Township has at Crumpsall a workhouse infirmary of 1,400 beds, and South Manchester at West Didsbury one of 1,039 beds. At Prestwich the infirmary, which is completely separated from the workhouse, is much smaller, containing only about 300 beds. The possibilities of reform arising from an amalgamation of this character in a compact and closely populated area are obvious, and it is to be hoped that the new Board of Guardians will take full advantage of them.

### TWO SERIOUS OMISSIONS.

It is only to be regretted that the Local Government Board have not had the courage to include Salford in the new Poor-Law area. The Local Government Board have also just issued a Provisional Order amalgamating the county boroughs of Plymouth and Devonport and the urban district of East Stonehouse for municipal purposes. The Provisional Order says nothing about the amalgamation of the corresponding Poor-Law areas, and it seems to be the general opinion locally that this omission implies that the Poor-Law areas are not to be amalgamated. This assumption may very possibly prove incorrect, and a separate Order may yet be issued amalgamating the Poor-Law areas also. The chief opposition to such amalgamation comes from the Devonport Guardians. The Plymouth Guardians support it strongly and have just passed a resolution that the non-inclusion of the Poor-Law administration in the amalgamation is viewed with grave misgivings and will be the subject of further deliberation by them. The advantages of amalgamation from the point of view of medical administration are so obvious that the Local Government Board can hardly overlook them.

### THE EARTHQUAKE AND THE DRUG MARKET.

The drug market has been more active during the past week, and considerable interest has been taken in several articles. Japanese refined camphor is again rather dearer, and an advance in the quotations of the English refined would not come as a surprise. Menthol is dearer, and a fair amount of business has been done; it is difficult to foresee the future of this market, but prices are by no means high notwithstanding the considerable advance that has recently taken place. Japanese dementholised peppermint oil has also had a dearer tendency. Contrary to first expectations, the recent earthquake in Sicily had practically no effect on the market for essence of lemon, quotations for which are unchanged; citric acid is again somewhat dearer, with no prospect of any decline in the immediate future. Cod-liver oil is slightly higher in price in consequence of the unfavourable results of the fishing in the Finmarken district; buyers, however, are not in a hurry. Opium is rather dearer, and morphine has an upward tendency in price. Quotations for chrysophanic acid are again higher as a result of the scarcity of Goa powder.

